# Halogen-Bond Assisted Photoinduced Electron Transfer

**DOI:** 10.3390/molecules24234361

**Published:** 2019-11-29

**Authors:** Bogdan Dereka, Ina Fureraj, Arnulf Rosspeintner, Eric Vauthey

**Affiliations:** Department of Physical Chemistry, University of Geneva, CH-1211 Geneva, Switzerland

**Keywords:** photochemistry, excited-state dynamics, time-resolved fluorescence, ultrafast IR spectroscopy

## Abstract

The formation of a halogen-bond (XB) complex in the excited state was recently reported with a quadrupolar acceptor–donor–acceptor dye in two iodine-based liquids (*J. Phys. Chem. Lett.* **2017**, *8*, 3927–3932). The ultrafast decay of this excited complex to the ground state was ascribed to an electron transfer quenching by the XB donors. We examined the mechanism of this process by investigating the quenching dynamics of the dye in the S_1_ state using the same two iodo-compounds diluted in inert solvents. The results were compared with those obtained with a non-halogenated electron acceptor, fumaronitrile. Whereas quenching by fumaronitrile was found to be diffusion controlled, that by the two XB compounds is slower, despite a larger driving force for electron transfer. A Smoluchowski–Collins–Kimball analysis of the excited-state population decays reveals that both the intrinsic quenching rate constant and the quenching radius are significantly smaller with the XB compounds. These results point to much stronger orientational constraint for quenching with the XB compounds, indicating that electron transfer occurs upon formation of the halogen bond.

## 1. Introduction

Although halogen bonds (XB) have been known of for a long time, their relevance to chemistry, biology and material sciences was realised relatively recently [1,2,3,4,5,6,7,8,9]. The halogen bond is often called the hydrophobic analogue of the hydrogen bond [5,10]. It originates from the attractive interaction between the electron-rich nucleophilic part of the XB accepting molecule and the so-called σ-hole; i.e., the region of depleted electron density located at the pole of the halogen atom participating in the polar covalent bond [11]. Although sharing some similarities with H-bonds, halogen bonds exhibit distinct differences: they are hydrophobic and more directional than hydrogen bonds [12], and their strength and length can be tuned by changing the nature of the halogen [13]. The exploitation of these features appears to be a powerful tool in various areas of molecular sciences, including supramolecular chemistry, catalysis and crystal engineering.

Up to now, the majority of XB studies have been carried out in the solid phase, where the bond can be easily identified using high-resolution X-ray diffraction on crystals. X-bonding interactions in the electronic excited state were considered in the context of solid-state, light-emitting materials [14,15,16]. In some cases, their main use was to enhance intersystem crossing to the triplet state via the heavy-atom effect [17]. In other cases, X-bonding interactions were exploited to tune the packing of chromophores by co-crystallising them with XB donors, and thus, to influence their fluorescence properties [18,19,20], or to enhance the self-assembly of supramolecular light-responsive polymers [21,22,23,24,25]. However, in all these cases, the halogen bonds themselves did not lead to intrinsically different excited-state properties of the chromophores. Significantly less experimental work was reported in liquid solution, and in all these cases, XB interactions in the electronic ground state were considered. In some of these cases, the XB complexes were shown to have a high photoreactivity involving bond cleavage and radical formation that could be exploited in organic synthesis [26,27,28]. In another case, XB formation was, on the contrary, found to inhibit photoinduced intramolecular electron transfer (ET) in a donor-acceptor dyad, enhancing its fluorescence [29]. XB interactions were also shown to play a beneficial role in the regeneration process of the photo-oxidised sensitiser in dye-sensitised-solar cells [30,31,32].

**Figure 1:**
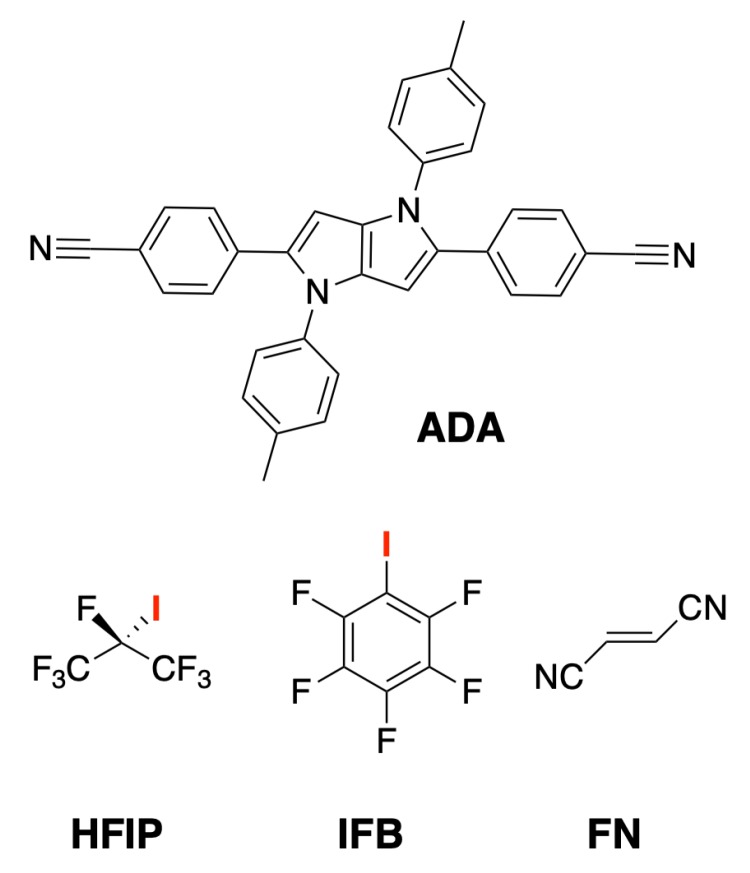
Structure of the quadrupolar dye **ADA** and of the three electron acceptors.

We recently reported on the observation of XB formation between an acceptor–donor–acceptor molecule (**ADA**, Figure 1) in the S_1_ electronic excited state and two iodine-based XB donors, perfluorinated isopropyl iodide (HFIP) and perfluorinated iodobenzene (IFB, Figure 1), acting as solvents, using time-resolved IR (TRIR) spectroscopy [33]. S_1_←S_0_ excitation of **ADA** in polar solvents was shown to be followed by so-called excited-state symmetry breaking (ES-SB); i.e., a transition from a quadrupolar Franck–Condon S_1_ state with an even distribution of the electronic excitation to a dipolar equilibrium S_1_ state with a higher electronic density on one of the two D-A branches [34]. As a consequence of ES-SB, the terminal N atom on the most excited branch is more basic, favouring the formation of H-bonds in protic solvents and halogen-bond in HFIP and IFB [33]. ES-SB of **ADA** was evidenced by the presence of two CN stretching absorption bands in the TRIR spectra instead of a single one as observed in non-polar solvents. The splitting of these two bands was shown to reflect the extent of ES-SB and to increase with solvent polarity [34,35]. H-bond and XB interactions were evidenced by the significantly larger band splitting than what was observed for non-protic or non-halogenated solvents of the same polarity. This larger splitting is consistent with an enhanced ES-SB due to the formation of a hydrogen or a halogen-bond on one side of **ADA**. XB formation was found to lead to a significant shortening of the excited-state lifetime of **ADA**, which was explained by the occurrence of an electron transfer (ET) between **ADA** in the S_1_ state and the two XB donors, acting as electron acceptors, followed by an ultrafast charge recombination [33].

Here, we report on a detailed investigation of the ET quenching dynamics of **ADA** by HFIP and IFB in two inert solvents of different polarities and a comparison with a non-halogenated electron acceptor, fumaronitrile (FN, Figure 1), using TRIR and time-resolved fluorescence spectroscopy. Although FN is a weaker electron acceptor than HFIP, ET is significantly faster with the former. Analysis of the quenching dynamics using the Smoluchowski–Collins–Kimball (SCK) model revealed that the slower quenching with XB electron acceptors can be explained in terms of more severe orientational constraints. This is consistent with the requirement of a XB to be formed for ET to be operative with the halogenated compounds. This is, to the best of our knowledge, the first detailed study of a halogen-bond assisted photoinduced ET reaction.

## 2. Results

**Figure 2:**
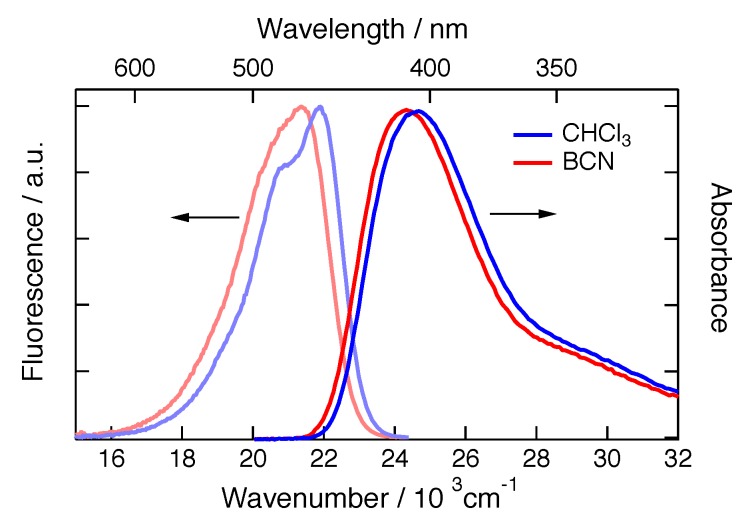
Electronic absorption and emission spectra of **ADA** in CHCl_3_ and BCN.

Figure 2 shows the absorption and fluorescence spectra of **ADA** in CHCl_3_ and benzonitrile (BCN). The absorption solvatochromism of **ADA** depends mostly on the refractive index of the solvent, pointing to dispersion as the dominant solute-solvent interaction in the ground state, in agreement with its symmetric, quadrupolar character. As discussed earlier [33,34], the larger shift of the fluorescence spectrum observed by going from the medium polar CHCl_3_ to the highly polar BCN reflects the dipolar character of the equilibrium S_1_ state as a consequence of ES-SB. The fluorescence quantum yields and lifetimes in CHCl_3_ and BCN are similar and are listed in Table 1.

The addition of HFIP or IFB has a negligible effect on the absorption spectrum apart from that due to refractive index changes. The S_1_←S_0_ absorption band of **ADA** in pure HFIP and IFB peaks at the same wavelength as that measured in a non X-bonding solvent of similar refractive index, suggesting negligible XB interactions in the electronic ground state [33]. On the other hand, emission is strongly quenched upon addition of HFIP and IFB in both CHCl_3_ and BCN, and is hardly detectable in pure HFIP and IFB.

**Figure 3:**
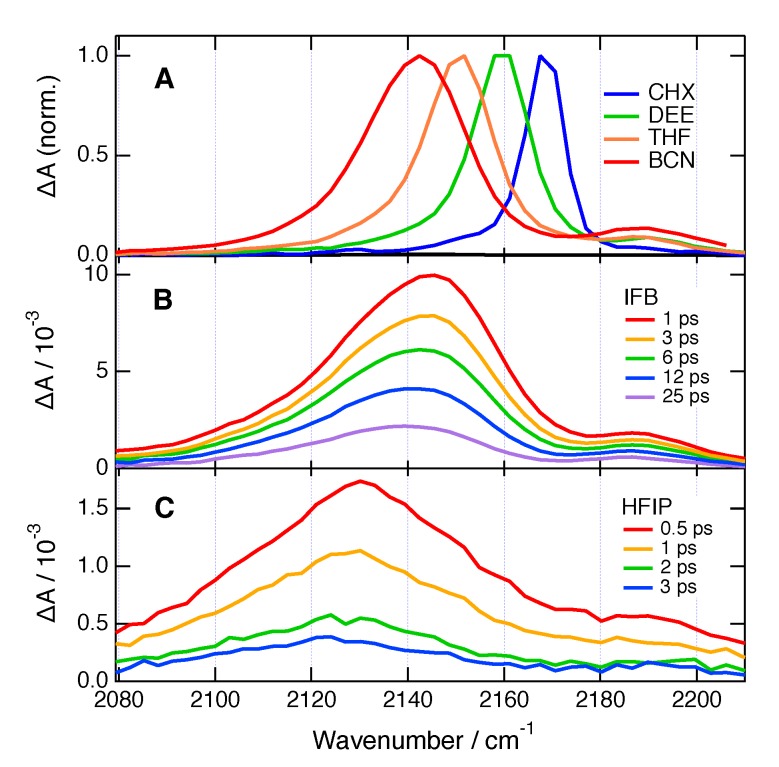
TRIR spectra recorded with **ADA** (**A**) in solvents of varying dielectric constants, $\varepsilon_{s}$ (CHX: cyclohexane ($\varepsilon_{s}$ = 2.0); DEE: diethyl ether ($\varepsilon_{s}$ = 4.3); THF: tetrahydrofuran ($\varepsilon_{s}$ = 7.6); BCN: benzonitrile ($\varepsilon_{s}$ = 25.2) at time delays longer than 100 ps, and (**B**) in HFIP and (**C**) IFB at different time delays after excitation.

The excited-state dynamics of **ADA** in pure solvents, including pure HFIP and IFB, were already reported earlier [33] and are briefly summarised here. Figure 3A shows TRIR spectra recorded in the CN stretching region with **ADA** in solvents of increasing polarity at sufficiently long time delay after excitation to correspond to the relaxed S_1_ excited state. In the non-polar cyclohexane, the spectrum consists of a single excited-state absorption (ESA) band due to the antisymmetric CN stretching mode. The presence of a single band is indicative of a symmetric S_1_ state. ES-SB takes place in all polar solvents. The resulting TRIR spectra exhibit an intense band (ESA1) due to the antisymmetric CN stretching mode, and a weak one (ESA2) originating from the symmetric CN stretching mode, which is no longer IR forbidden. The increasing band splitting and relative intensity of ESA2 with solvent polarity reflect the increasing extent of ES-SB.

Figure 3B,C shows TRIR spectra in the same frequency region measured with **ADA** in pure IFB and HFIP. The temporal increase of band splitting is clearly visible in IFB and reflects the equilibration of the S_1_ state. Because of the short excited-state lifetime in IFB and especially in HFIP, the TRIR spectra at the longest time delay shown here do probably not correspond to the fully equilibrated S_1_ state of **ADA**. Despite this, the width of ESA1, the band splitting and the relative intensity of ESA2 in these solvents are significantly larger than in non-halogenated solvents of the same polarity and are similar to those found in protic solvents where H-bonding is operative [33,34]. These effects reflect the occurrence of a specific solute–solvent interaction; namely, X-bonding. The time-dependent shift of ESA1 reflects the formation of the halogen bond on the branch of **ADA** that bears most excitation and its equilibration upon reorientation of the surrounding solvent molecules, as discussed in detail in [33]. The excited-state lifetime of **ADA**, extracted from the analysis of the TRIR dynamics, amounts to 1.5 ps in HFIP and 12 ps in IFB, pointing to a strong quenching in these two solvents. In HFIP, the decay of ESA1 and ESA2 is accompanied by a full recovery of the ground-state population, as evidenced by the concomitant disappearance of the ground-state bleach. By contrast, in IFB, a small residual bleach remains after the full decay of ESA1 and ESA2. Additionally, the residual spectra show a very weak positive band at 2185 cm^−1^, that was attributed to **ADA** in the triplet state [33]. The population of the T_1_ state is due to intersystem crossing (ISC) from **ADA** in the S_1_ state enhanced by the external heavy-atom effect. A triplet yield of 7% can be estimated from the relative amplitude of the residual bleach. Similar dynamics were observed by transient electronic absorption (TA) measurements [33]. No transient species other than the S_1_ and the T_1_ states of **ADA** was observed in either TRIR and TA spectra. The quenching was attributed to an ET from **ADA** in the S_1_ state to HFIP and IFB, and the absence of quenching product bands was explained by an ultrafast charge recombination of the ensuing radical ion pair.

To investigate this ET quenching in more detail, TRIR measurements were performed in CHCl_3_ and BCN with varying concentrations of HFIP and IFB. The resulting spectra exhibit the same two ESA bands, at positions intermediate to those measured without quencher and those in pure quencher solvents. These spectra display little dynamics apart from the decay of the ESA and bleach bands. No other transient than the S_1_ state can be observed. No triplet band could be detected with IFB. This indicates that ISC can only compete with ET quenching if the external heavy-atom effect is strong enough. This condition is apparently only fulfilled in pure IFB.

**Figure 4:**
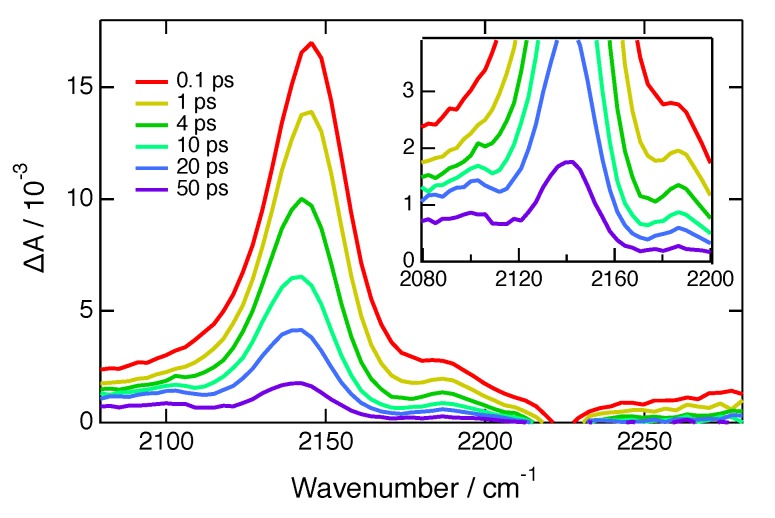
TRIR spectra recorded at different time delays after excitation of **ADA** in CHCl_3_ with 1 M FN. The inset is a zoom in the 2100 cm^−1^ band region.

Figure 4 depicts TRIR spectra recorded with **ADA** in CHCl_3_ in the presence of 1 M FN. Here, contrary to HFIP and IFB, the decay of ESA1 and ESA2 is accompanied by the appearance of a new ESA band at 2100 cm^−1^. This frequency is very similar to that of the CN stretch band of the reduced 1,4-dicyanobenzene [36] and is, thus, ascribed to the radical anion of FN. By analogy to the radical cation of 9,10-dicyanoanthracene [37], the CN stretch band of the oxidised **ADA** is probably too weak to be observed. The 2100 cm^−1^ band of FN^−^ decays entirely with a ∼40 ps time constant and is only visible at large FN concentrations where quenching is fast enough. The absence of any ET product band with HFIP and IFB as well as the full ground-state recovery suggest that charge recombination is faster with these two halogenated quenchers than with FN.

**Figure 5:**
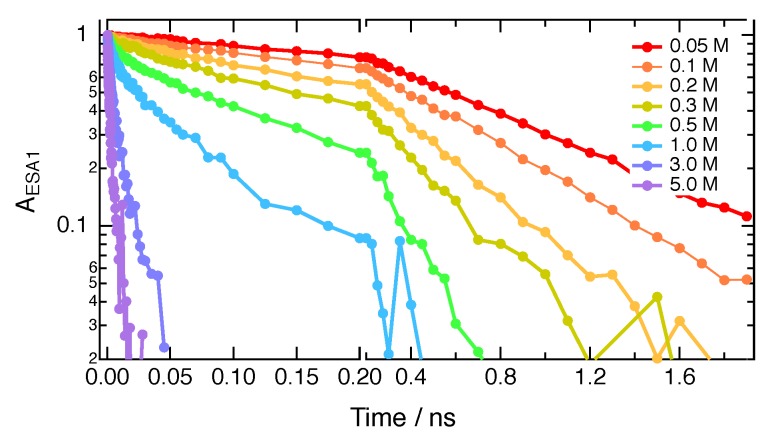
Time evolution of the excited-state population of **ADA** (area of ESA1) in CHCl_3_ with different concentrations of HFIP.

To extract the time evolution of the population of **ADA** in the S_1_ state from these data, the area of ESA1 was determined from a band-shape analysis of the TRIR spectra using the sum of three Gaussian functions (ESA1, ESA2 and bleach). Figure 5 shows the time evolution of the area of ESA1 in CHCl_3_ in the presence of various HFIP concentrations. Similar data obtained in BCN and with the other quenchers are shown in the Supporting Information (Figures S1–S5).

Given the relatively narrow time-window of the TRIR experiments (0–2 ns), the full quenching dynamics could only be recorded at relatively high quencher concentrations (>0.3 M). The dynamics at lower quencher concentrations were investigated using time-resolved fluorescence. As the instrument response function of the time-correlated single photon counting (TCSPC) setup was about 200 ps, the fluorescence time-profiles mostly reflect the decay of the excited-state population and not the faster spectral dynamics associated with ES-SB and solvent relaxation.

**Figure 6:**
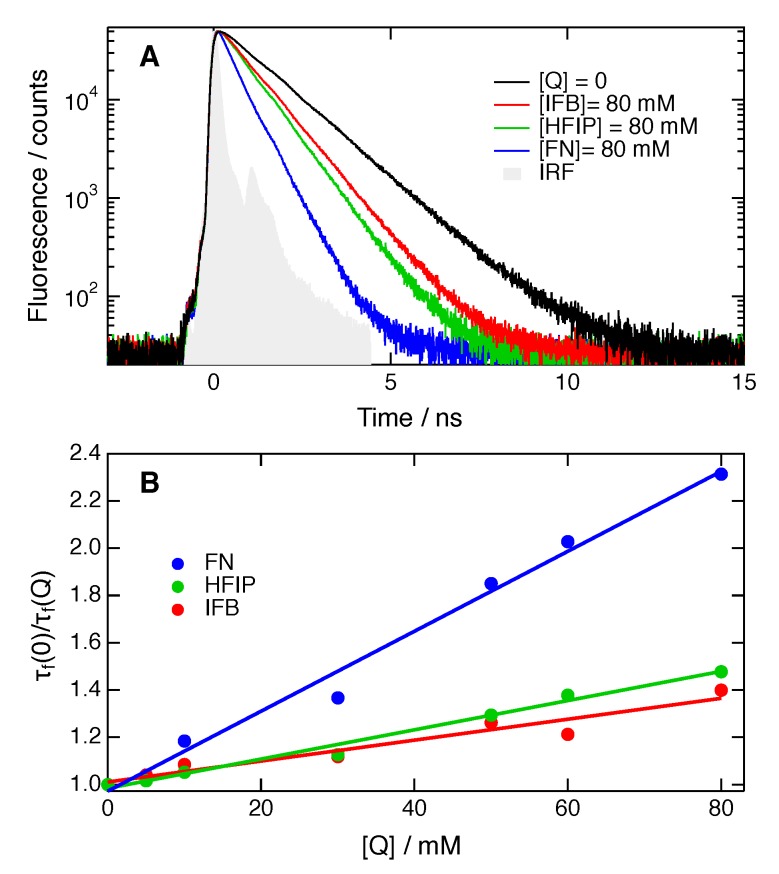
(**A**) Fluorescence time profiles measured with **ADA** in CHCl_3_ alone and in the presence of 0.08 M HFIP, IFB and FN. (**B**) Stern–Volmer plots of the fluorescence quenching of **ADA** by HFIP, IFB and FN in CHCl_3_.

Figure 6A and Figures S6–S8 reveal that, for a given quencher concentration in CHCl_3_, the excited-state decay becomes faster by going from IFB to HFIP and to the non-halogenated FN. The same behaviour is observed in BCN (Figures S9–S11). The fluorescence time profiles could be well reproduced by the convolution of the instrument response function with a single exponential decay.

However, the TRIR decays measured with higher quencher concentrations are non-exponential (Figure 5), as expected for a diffusion-assisted ET quenching process, when measured with a sufficiently high time resolution [38,39,40,41,42]. This is due to the different stages of the quenching; namely, the static, the transient and the stationary stages. Directly after excitation, quenching is static as it occurs with reactant pairs at near-optimal distance and mutual orientation, and thus, does not require any diffusion. Once these pairs have reacted, quenching takes place with pairs at sub-optimal distance/orientation and slows down with time (transient stage). This slowing down occurs until the rates of mutual approach of the reactants via diffusion and their disappearance upon quenching have equilibrated. As a consequence, the ET rate is time dependent and decreases from $k_{0}$ in the short-time (static) limit to $k_{\infty }$ in the long-time (stationary) limit.

Several approaches can be applied to analyse the quenching dynamics and to extract $k_{0}$ and $k_{\infty }$, one of the most advanced being the encounter theory [41,43]. It accounts for both the time dependence of the reactant-pair distribution and the distance dependence of the ET reaction within the framework of Marcus theory. However, to be really insightful, this approach requires knowledge of a relatively large number of ET parameters, among which the driving force and the reorganisation energy. Without this information, the number of adjustable parameters is too large for such an analysis to give unambiguous results.

As several ET parameters of **ADA** and of the quenchers are not known precisely, $k_{0}$ was obtained by analysing the quenching dynamics measured by TRIR using the Smoluchowski–Collins–Kimball (SCK) model, whereas $k_{\infty }$ was determined by a Stern–Volmer analysis of the slow quenching dynamics measured by time-resolved fluorescence at low quencher concentrations:

(1) $$\frac{\tau_{f}\left(0\right)}{\tau_{f}\left(Q\right)}=1+k_{\infty }\tau_{f}\left(0\right)\left[Q\right],$$

where $\tau_{f}\left(0\right)$ and $\tau_{f}\left(Q\right)$ are the fluorescence lifetimes without quencher and at quencher concentration [Q], respectively. The Stern–Volmer plots obtained with all three quenchers in both solvents at [*Q*] < 0.1 M, are linear, as shown in Figure 6B and Figure S12. The $k_{\infty }$ values obtained from a linear fit of Equation (1) are listed in Table 2.

The SCK model of diffusion-controlled reactions is a variant of that of Smoluchowski. In both models, one of the reactants (i.e., the excited one) is at a fixed position at the centre of a reaction sphere of radius *R* and the other (the quencher) diffuses with the mutual diffusion coefficient of the two reactants, *D*. In the Smoluchowski model [44,45,46], the reaction occurs instantaneously as soon as the diffusing reactant penetrates the reaction sphere. In the SCK model [45,46,47], the reaction has a finite rate constant, $k_{0}$. The main difference between the SCK model and the encounter theory is that the ET rate is not distance dependent in the former.

According to the SCK model, the time evolution of the excited-state population is [38,48,49]:

(2) $$P\left(t\right)=P\left(0\right)\exp\left[-\left(\tau_{0}^{-1}+p\right)t-\frac{q}{c^{2}}\left(\exp\left(c^{2}t\right)\mathrm{erfc}\left(ct^{1/2}\right)-1+\frac{2ct^{1/2}}{\pi^{1/2}}\right)\right],$$

where P(0) is the excited-state population at t=0 after excitation, $\tau_{0}$ is the excited-state lifetime in the absence of quencher, p=a[Q], q=b[Q] and:

(3) $$\begin{array}{c} a=k_{0}{\left(1+\frac{k_{0}}{4\pi RDN_{A}}\right)}^{-1}, \end{array}$$

(4) $$\begin{array}{c} b=k_{0}{\left(1+\frac{4\pi RDN_{A}}{k_{0}}\right)}^{-1}, \end{array}$$

(5) $$\begin{array}{c} c=\left(1+\frac{k_{0}}{4\pi RDN_{A}}\right)\frac{D^{1/2}}{R}, \end{array}$$

with Avogadro’s number $N_{A}$. In principle, $k_{0}$, *R* and *D* can be obtained from the fit of Equation (2) to the TRIR excited-state population decays. However, as *R* and *D* are closely associated, good fits of Equation (2) could be obtained with different pairs of *R* and *D* values. In order to eliminate the ambiguity on these two parameters, the diffusion coefficients of **ADA** and of the three quenchers were determined using NMR diffusion-ordered spectroscopy (DOSY, see Supporting Information for details).

**Figure 7:**
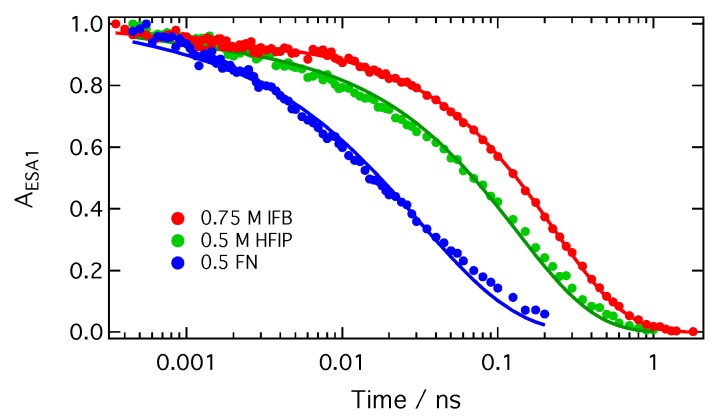
Best-fits of Equation (2) to the excited-state decays of **ADA** in CHCl_3_ in the presence of HFIP, IFB and FN.

SCK analysis was only performed with TRIR time profiles measured at large enough quencher concentrations for the population decay to be complete within the temporal window of the experiments. Furthermore, this analysis was not carried out when the population decay was complete in less than about 100 ps. In such cases, occurring mostly with HFIP concentrations larger than about 1 M in CHCl_3_, quenching is so fast that some early ultrafast decay components might be missed with the 200–300 fs response function of the experiment. Moreover, excluded volume effects become important at high quencher concentrations and render analytical theories inaccurate [50]. Good fits to the data were obtained with HFIP and IFB in both solvents within the range of concentrations specified in Table 2 and using the experimental diffusion coefficients (Figure 7 and Figures S1–S4). The fits to the **ADA**/FN data with the experimental *D* values were not as satisfactory (Figure 7 and Figure S5). Such deviations have already been observed in the SCK analysis of fluorescence quenching by intermolecular ET [51,52,53,54] and were attributed to the neglect of the distance dependence of ET in the SCK model. The best-fit parameters are listed in Table 2.

## 3. Discussion

The following trends can be deduced from Table 2:

(i) The intrinsic ET rate constant, $k_{0}$, decreases in the order, FN, HFIP, IFB, in the same way as $k_{\infty }$.
(ii) For a given quencher, $k_{0}$ is larger or equal in BCN than in CHCl_3_, contrary to $k_{\infty }$ which is smaller in BCN.
(iii) The quenching radius, *R*, is significantly larger for FN than for the halogenated acceptors.
(iv) For a given quencher, *R* is larger in BCN than in CHCl_3_.

In order to rationalise these trends, we have first to consider the ET driving force [55]:

(6) $$\Delta G_{\mathrm{ET}}=-E_{S_{1}}-e\left[E_{\mathrm{red}}\left(A\right)-E_{\mathrm{ox}}\left(D\right)\right]+C+S,$$

where $E_{S_{1}}$ is the energy of **ADA** in the S_1_ state; $E_{\mathrm{ox}}\left(D\right)$ and $E_{\mathrm{red}}\left(A\right)$ are the oxidation and reduction potentials of **ADA** and the quenchers, respectively; and *C* and *S* are corrections factors that account for the Coulombic interactions in the quenching product and for a solvent different from that used for the determination of the redox potentials, respectively. In a polar solvent like BCN, *C* and *S* can be neglected. Taking $E_{S_{1}}$ = 2.85 eV, $E_{\mathrm{ox}}\left(\mathrm{ADA}\right)$ = 1.22 V versus SCE as determined by cyclic voltammetry (Figure S13), and literature values for the reduction potential of HFIP (−1.0 V versus SCE [56]) and FN (−1.36 V versus SCE [57]), gives $\Delta G_{\mathrm{ET}}=$−0.62 and −0.3 eV for the quenching by HFIP and FN in BCN. No literature value could be found for the reduction potential of IFB. In the less polar CHCl_3_, the ET driving forces can be estimated to be less exergonic than in BCN by about 0.25 eV.

According to Marcus ET theory [58], this range of moderate exergonicity corresponds to the normal region, where the ET rate constant is predicted to increase with driving force. Therefore, the observed increase of $k_{0}$ by going from CHCl_3_ to BCN (trend ii), is totally consistent. On the other hand, the smaller $k_{\infty }$ values measured in BCN can be accounted for by the higher viscosity of this solvent, which slows down diffusion. This can be seen by considering the diffusion rate constants, $k_{d}$, calculated assuming reactants of the same size (Table 1).

The significantly smaller $k_{0}$ measured with IFB compared to HFIP in both solvents can probably be accounted for by a smaller driving force. Although its reduction potential is not known, IFB can be expected to be a weaker acceptor than HFIP, based on the fact that the σ-hole in IFB is significantly smaller and less positive than that in HFIP [33].

However, the smaller $k_{0}$ found with HFIP relative to FN (trend i), despite a significantly larger driving force, is unexpected. It points to the involvement of another factor that affects ET and should be markedly different for FN and the halogenated quenchers.

Apart from the driving force, the parameters in Marcus theory that affect the ET rate constant are the reorganisation energy, λ, and the electronic coupling, *V* [58]. In principle, a difference in reorganisation energy between HFIP and FN larger than the difference in driving force, i.e., larger than 0.3 eV, could account for the smaller $k_{0}$ found with HFIP. This difference should mostly arise from the intramolecular reorganisation energy, $\lambda_{i}$. However, considering that $\lambda_{i}$ is usually of the order of 0.3–0.4 eV for ET between organic molecules [59,60], a >0.3 eV increase due to only one of the two reactants would imply an unusually large reorganisation energy for the reduction of HFIP. Although this cannot be totally excluded, this does not seem highly probable.

The smaller $k_{0}$ found with HFIP is most likely associated with *V*. The magnitude of the electronic coupling depends not only on the distance between the reactants but also on their mutual orientation [61,62]. Indeed, *V* is not isotropic, unless the reactants are spherical. In the case of FN, a relatively large distribution of orientations relative to **ADA** can be expected to give a significant π-orbital overlap [63]. For HFIP and IFB, *V* can be anticipated to be noticeably more anisotropic, because coupling requires a well defined orientation of the iodine atom, i.e., of the σ-hole, relatively to one of the CN ends of **ADA**. Therefore, the orientation-averaged value of *V* should be significantly smaller for the XB quenchers than for FN [64,65].

This explanation is consistent with trend iii; i.e., the smaller quenching radius *R* found for HFIP and IFB compared to FN. Given the strongly non-spherical shape of **ADA**, this radius cannot be considered literally but should reflect the anisotropy of *V*. Indeed, the quenching radius for a reactant pair for which any mutual orientation gives a significant coupling should be much larger than that for a reactant pair with severe orientational constraints [64,65]. The smaller σ-hole of IFB compared to HFIP implies larger orientational constraints for IFB, and thus, results in a smaller *R* value in the SCK analysis. This is also consistent with a smaller ET driving force with IFB. In general, the penalty due to a weak driving force can be compensated by a larger electronic coupling. In the case of IFB, this translates into stronger orientational constraints; hence a smaller *R*.

The same argument explains trend iv; i.e., the smaller *R* values in CHCl_3_ compared to BCN found with both XB quenchers. The weaker ET driving force in the less polar CHCl_3_ requires larger *V* for ET to be operative, and thus a smaller *R*.

**Figure 8:**
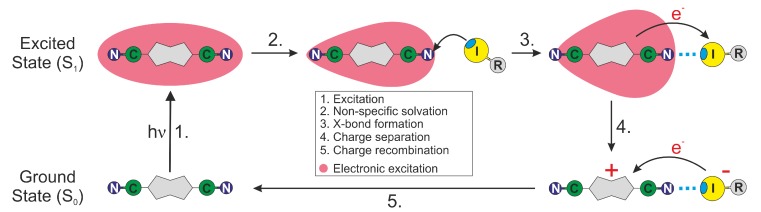
Schematic representation of the photocycle of **ADA** in the presence of a XB donor. Excited-state symmetry breaking is represented by the change of shape of the red-shaded area.

In summary, all the above trends in the SCK parameters obtained from the analysis of the TRIR decays are consistent with an ET between **ADA** in the S_1_ state and the halogenated quenchers occurring predominantly through XB interaction (Figure 8). This interaction leads to a mutual orientation of the reactants that enable sufficient electronic coupling. As a consequence, the excited XB complex is very short lived because ET occurs rapidly upon its formation. This ET mechanism via a halogen bond explains why the HFIP and IFB data can be reproduced well within the SCK model, which assumes that quenching only takes place when the reactants are at a fixed distance *R*. For the same reason, the dynamics of bimolecular excited-state proton transfer reactions could be successfully reproduced in terms of the SCK model [66,67,68]. ET quenching of **ADA** by HFIP and IFB without XB formation cannot be excluded, but can be expected to be much less efficient.

## 4. Materials and Methods

### 4.1. Chemicals

2,6-di(4-cyanophenyl)-1,5-di(4-methylphenyl)-3,4-dihydropyrrolo[3,2-b]pyrrole, **ADA**, was provided by D. Gryko from the Institute of Organic Chemistry of the Polish Academy of Sciences in Warsaw and synthesised as described in [69]. All solvents and quenchers were purchased from Sigma-Aldrich or Acros Organics, were of 99.0% or higher purity, and were used as received.

### 4.2. Stationary Spectroscopy

Electronic absorption spectra were recorded on a Cary 50 spectrometer, whereas fluorescence spectra were measured on a FluoroMax-4 (Jobin Yvon) and corrected using a set of secondary emissive standards [70].

### 4.3. DOSY NMR

The DOSY NMR measurements were performed at 25 °C on a Bruker 500 MHz ^1^H NMR Larmor frequency spectrometer equipped with a DCH helium-cooled detection probe and a z-gradient coil with a maximum nominal gradient strength of 65 G cm^−1^. The same procedure as described in [71] was applied.

### 4.4. Time-Resolved Fluorescence

Sub-nanosecond time-resolved fluorescence dynamics were measured using the time-correlated single photon counting (TCSPC) technique with the setup described in [72]. Excitation was carried out with a laser diode at 395 nm (LDH-P-C-400B, PicoQuant). The pulse duration was 60 ps and the full width at half maximum (FWHM) of the instrument response function was about 200 ps. The fluorescence was collected at magic angle relative to the polarisation of the excitation pulse and passed through an interference filter with 8 nm bandwidth centred at 450 nm.

### 4.5. Time-Resolved Infrared Spectroscopy

Time-resolved IR (TRIR) measurements were carried out with the same setup as described in [33,37]. Excitation was achieved with 400 nm pulses generated by frequency doubling part of the output of a 1 kHz amplified Ti:Sapphire system (Solstice, Spectra-Physics). Probing was achieved with the output of an optical parametric amplifier (TOPAS-C, Light Conversion) connected to a difference-frequency mixing module (NDFG, Light Conversion) and polarised at magic angle relative to the pump pulse. Detection was performed with a 2 × 64 element MCT array (Infrared Systems Development) connected with a spectrograph (Triax190, 150 lines per mm, Horiba), resulting in a spectral resolution of 1.5 cm^−1^. Sample handling and data acquisitions were the same as described in [33].

## 5. Conclusions

The results presented here evidence that the ET quenching of **ADA** by HFIP and IFB proceeds with a different mechanism than the quenching by a conventional non-halogenated electron acceptor like FN. Despite larger driving force, the quenching by HFIP is significantly slower than with FN. Moreover, SCK analysis of the quenching dynamics points to a markedly shorter reaction radius than for FN. These differences can be explained by much stronger restrictions on the mutual orientation of the reactants for the halogenated quenchers. For quenching to be efficient, the iodine atom has to point towards one of the CN ends of **ADA**, whereas a much broader distribution of orientations permits a large coupling with FN. The orientations of the halogenated quencher where coupling to **ADA** is significant are the same as those which favour XB interactions.

Such a strong connection between halogen bond and ET implies that XB formation with an excited molecule should always be followed by an ET. Because of this, excited XB complexes can be expected to be generally short lived. This halogen-bond assisted ET quenching observed here with a quadrupolar A–D-A molecule should also occur with push-pull D-A dyes provided the electronic density on the A moiety in the excited state is sufficient to ensure strong electrostatic interactions with the σ-hole of the XB donor. The ET product could not be observed here due to ultrafast charge recombination to the neutral ground state. Further investigations are needed to find out whether such fast recombination is also a general feature of halogen-bond assisted ET or whether recombination can be slow enough for dissociation into free ions to be operative.

## Figures and Tables

**Table 1:** Solvent properties (dielectric constant, $\varepsilon_{s}$; diffusion rate constant assuming reactants of the same size, $k_{d}$); fluorescence quantum yields, $\Phi_{f}$; and excited-state lifetimes, $\tau_{S1}$, of **ADA**. The dielectric constant of HFIP is not known but should be between those of 2-iodopropane ($\varepsilon_{s}=8.2$) and 1,1,1,3,3,3-hexafluoro-2-methoxypropane ($\varepsilon_{s}=14.8$).

Solvent	$\varepsilon_{s}$	$k_{d}$/M^−1^ns^−1^	$\Phi_{f}$	$\tau_{S1}$
CHCl_3_	4.8	12	0.78	1.3 ns
BCN	25.2	5.4	0.81	1.4 ns
HFIP	8–14			1.5 ps
IFB	5.6			12 ps

**Table 2:** Rate constants, $k_{0}$ and $k_{\infty }$, and quenching radii, *R*, obtained from a Stern–Volmer and SCK analysis of the quenching dynamics of **ADA**, and mutual diffusion coefficients, *D*, within the range of quencher concentrations used for the SCK analysis.

Quencher	Solvent	$k_{\infty }$ /M^−1^ns^−1^	$k_{0}$ /M^−1^ns^−1^	*R* Å	[*Q*] M	*D* Å^2^ ns^−1^
HFIP	CHCl_3_	4.7 ± 0.2	150 ± 30	4.7 ± 0.2	0.4–1.0	246–242
IFB		3.4 ± 0.4	20 ± 13	3.5 ± 0.3	0.3–3.0	210–175
FN		12.8 ± 0.6	220 ± 20	9.0 ± 1.0	0.1–1.0	260–240
HFIP	BCN	3.3 ± 0.4	150 ± 25	6.0 ± 0.3	0.1–0.5	107–110
IFB		3.3 ± 0.2	25 ± 15	5.0 ± 0.2	0.3–3.0	93–85
FN		6.9 ± 0.4	–	–		–

## Supplementary Materials

The following are available online: additional TRIR and time-resolved fluorescence data, electrochemistry and diffusion coefficients. The experimental data can be downloaded from http://doi.org/10.5281/zenodo.3528207.

## Abbreviations

The following abbreviations are used in this manuscript:

BCN	Benzonitrile
DOSY	Diffusion Ordered SpectroscopY
ESA	Excited-State Absorption
ES-SB	Excited-State Symmetry Breaking
ET	Electron Transfer
FN	Fumaronitrile
HFIP	Perfluoroisopropyl iodide
IFB	Iodoperfluorobenzene (pentafluoroiodobenzene)
ISC	InterSystem Crossing
SCK	Smoluchowski–Collins–Kimball (model)
TA	Transient Absorption
TCSPC	Time-Correlated Single Photon Counting
TRIR	Time-Resolved IR spectroscopy
XB	Halogen bond

## References

[1] Metrangolo P., Resnati G. (2008). Halogen versus Hydrogen. Science.

[2] Cavallo G., Metrangolo P., Pilati T., Resnati G., Sansotera M., Terraneo G. (2010). Halogen bonding: A general route in anion recognition and coordination. Chem. Soc. Rev..

[3] Scholfield M.R., Vander Zanden C.M., Carter M., Ho P.S. (2013). Halogen bonding (X-bonding): A biological perspective. Protein Sci..

[4] Gilday L.C., Robinson S.W., Barendt T.A., Langton M.J., Mullaney B.R., Beer P.D. (2015). Halogen Bonding in Supramolecular Chemistry. Chem. Rev..

[5] Cavallo G., Metrangolo P., Milani R., Pilati T., Priimagi A., Resnati G., Terraneo G. (2016). The Halogen Bond. Chem. Rev..

[6] Mendez L., Henriquez G., Sirimulla S., Narayan M. (2017). Looking back, looking forward at halogen bonding in drug discovery. Molecules.

[7] Tepper R., Schubert U.S. (2018). Halogen Bonding in Solution: Anion Recognition, Templated Self-Assembly, and Organocatalysis. Angew. Chem. Int. Ed..

[8] Juanes M., Saragi R.T., Caminati W., Lesarri A. (2019). The Hydrogen Bond and Beyond: Perspectives for Rotational Investigations of Non-Covalent Interactions. Chem. Eur. J..

[9] Saccone M., Catalano L. (2019). Halogen Bonding beyond Crystals in Materials Science. J. Phys. Chem. B.

[10] Priimagi A., Cavallo G., Metrangolo P., Resnati G. (2013). The Halogen Bond in the Design of Functional Supramolecular Materials: Recent Advances. Acc. Chem. Res..

[11] Clark T., Hennemann M., Murray J.S., Politzer P. (2007). Halogen Bonding: The *σ*-Hole. J. Mol. Model..

[12] Politzer P., Murray J.S., Clark T. (2010). Halogen Bonding: An Electrostatically-Driven Highly Directional Noncovalent Interaction. Phys. Chem. Chem. Phys..

[13] Riley K.E., Murray J.S., Fanfrlík J., Řezáč J., Solá R.J., Concha M.C., Ramos F.M., Politzer P. (2011). Halogen Bond Tunability I: The Effects of Aromatic Fluorine Substitution on the Strengths of Halogen-Bonding Interactions Involving Chlorine, Bromine, and Iodine. J. Mol. Model..

[14] Zhu W., Zheng R., Zhen Y., Yu Z., Dong H., Fu H., Shi Q., Hu W. (2015). Rational design of charge-transfer interactions in halogen-bonded co-crystals toward versatile solid-state optoelectronics. J. Am. Chem. Soc..

[15] Liu R., Wang H., Jin W.J. (2017). Soft-Cavity-type Host-Guest Structure of Cocrystals with Good Luminescence Behavior Assembled by Halogen Bond and Other Weak Interactions. Cryst. Growth Des..

[16] Salunke J.K., Durandin N.A., Ruoko T.-P., Candeias N.R., Vivo P., Vuorimaa-Laukkanen E., Laaksonen T., Priimagi A. (2018). Halogen-Bond-Assisted Photoluminescence Modulation in Carbazole-Based Emitter. Sci. Rep..

[17] Shi L., Liu H.-Y., Shen H., Hu J., Zhang G.-L., Wang H., Ji L.-N., Chang C.-K., Jiang H.-F. (2009). Fluorescence properties of halogenated mono-hydroxyl corroles: the heavy-atom effects. J. Porphyr. Phthalocyanines.

[18] Yan D., Delori A., Lloyd G.O., Friščić T., Day G.M., Jones W., Lu J., Wei M., Evans D.G., Duan X. (2011). A Cocrystal Strategy to Tune the Luminescent Properties of Stilbene-Type Organic Solid-State Materials. Angew. Chem. Int. Ed..

[19] Botta C., Cariati E., Cavallo G., Dichiarante V., Forni A., Metrangolo P., Pilati T., Resnati G., Righetto S., Terraneo G. (2014). Fluorine-induced J-aggregation enhances emissive properties of a new NLO push–pull chromophore. J. Mater. Chem. C.

[20] Sun C.-L., Li J., Geng H.-W., Li H., Ai Y., Wang Q., Pan S.-L., Zhang H.-L. (2013). Understanding the Unconventional Effects of Halogenation on the Luminescent Properties of Oligo(Phenylene Vinylene) Molecules. Chem. Asian J..

[21] Priimagi A., Cavallo G., Forni A., Gorynsztejn–Leben M., Kaivola M., Metrangolo P., Milani R., Shishido A., Pilati T., Resnati G. (2012). Halogen Bonding versus Hydrogen Bonding in Driving Self-Assembly and Performance of Light-Responsive Supramolecular Polymers. Adv. Funct. Mater..

[22] Bushuyev O.S., Tomberg A., Friščić T., Barrett C.J. (2013). Shaping Crystals with Light: Crystal-to-Crystal Isomerization and Photomechanical Effect in Fluorinated Azobenzenes. J. Am. Chem. Soc..

[23] Bushuyev O.S., Corkery T.C., Barrett C.J., Friščić T. (2014). Photo-mechanical azobenzene cocrystals and in situ X-ray diffraction monitoring of their optically-induced crystal-to-crystal isomerisation. Chem. Sci..

[24] Saccone M., Dichiarante V., Forni A., Goulet-Hanssens A., Cavallo G., Vapaavuori J., Terraneo G., Barrett C.J., Resnati G., Metrangolo P. (2015). Supramolecular hierarchy among halogen and hydrogen bond donors in light-induced surface patterning. J. Mater. Chem. C.

[25] Virkki M., Tuominen O., Forni A., Saccone M., Metrangolo P., Resnati G., Kauranen M., Priimagi A. (2015). Halogen bonding enhances nonlinear optical response in poled supramolecular polymers. J. Mater. Chem. C.

[26] Sun X., Wang W., Li Y., Ma J., Yu S. (2016). Halogen-Bond-Promoted Double Radical Isocyanide Insertion under Visible-Light Irradiation: Synthesis of 2-Fluoroalkylated Quinoxalines. Org. Lett..

[27] Wang H., Wang W., Jin W.J. (2016). *σ*-Hole Bond vs *π*-Hole Bond: A Comparison Based on Halogen Bond. Chem. Rev..

[28] Li Y., Lu Y., Mao R., Li Z., Wu J. (2017). A photoinduced reaction of perfluoroalkyl halides with 1,3-diarylprop-2-yn-1-ones catalyzed by DABSO. Org. Chem. Front..

[29] Zou W.-S., Lin S., Li J.-Y., Wei H.-Q., Zhang X.-Q., Shen D.-X., Qiao J.-Q., Lian H.-Z., Xie D.-Q., Ge X. (2015). Mechanism and application of halogen bond induced fluorescence enhancement and iodine molecule cleavage in solution. New J. Chem..

[30] Tuikka M., Hirva P., Rissanen K., Korppi-Tommola J., Haukka M. (2011). Halogen bonding—A key step in charge recombination of the dye-sensitized solar cell. Chem. Commun..

[31] Simon S.J.C., Parlane F.G., Swords W.B., Kellett C.W., Du C., Lam B., Dean R.K., Hu K., Meyer G.J., Berlinguette C.P. (2016). Halogen Bonding Promotes Higher Dye-Sensitized Solar Cell Photovoltages. J. Am. Chem. Soc..

[32] Parlane F.G.L., Mustoe C., Kellett C.W., Simon S.J., Swords W.B., Meyer G.J., Kennepohl P., Berlinguette C.P. (2017). Spectroscopic detection of halogen bonding resolves dye regeneration in the dye-sensitized solar cell. Nat. Commun..

[33] Dereka B., Vauthey E. (2017). Solute–Solvent Interactions and Excited-State Symmetry Breaking: Beyond the Dipole–Dipole and the Hydrogen-Bond Interactions. J. Phys. Chem. Lett..

[34] Dereka B., Rosspeintner A., Krzeszewski M., Gryko D.T., Vauthey E. (2016). Symmetry-Breaking Charge Transfer and Hydrogen Bonding: Toward Asymmetrical Photochemistry. Angew. Chem. Int. Ed..

[35] Ivanov A.I., Dereka B., Vauthey E. (2017). A Simple Model of Solvent-Induced Symmetry-Breaking Charge Transfer in Excited Quadrupolar Molecules. J. Chem. Phys..

[36] Mohammed O.F., Banerji N., Lang B., Nibbering E.T., Vauthey E. (2006). Photoinduced bimolecular electron transfer investigated by femtosecond time-resolved infrared spectroscopy. J. Phys. Chem. A.

[37] Koch M., Letrun R., Vauthey E. (2014). Exciplex Formation in Bimolecular Photoinduced Electron-Transfer Investigated by Ultrafast Time-Resolved Infrared Spectroscopy. J. Am. Chem. Soc..

[38] Eads D.D., Dismer B.G., Fleming G.R. (1990). A subpicosecond, subnanosecond and steady-state study of diffusion influenced fluorescence quenching. J. Chem. Phys..

[39] Dorfman R.C., Fayer M.D. (1992). The influence of diffusion on photoinduced electron transfer and geminate recombination. J. Chem. Phys..

[40] Murata S., Tachiya M. (1996). Electron transfer reactions studied through the transient effect in fluorescence quenching. J. Chem. Phys..

[41] Burshtein A.I. (2004). Non-Markovian Theories of Transfer Reactions in Luminescence and Chemiluminescence and Photo- and Electrochemistry. Adv. Chem. Phys..

[42] Rosspeintner A., Vauthey E. (2014). Bimolecular Photoinduced Electron Transfer Reactions in Liquids under the Gaze of Ultrafast Spectroscopy. Phys. Chem. Chem. Phys..

[43] Burshtein A. (2000). Unified Theory of Photochemical Charge Separation. Adv. Chem. Phys..

[44] Von Smoluchowski M. (1918). Versuch einer mathematischen Theorie der Koagulationskinetik kolloider Loesungen. Z. Phys. Chem..

[45] Rice S.A. (1985). Diffusion Limited Reactions.

[46] Keizer J. (1987). Diffusion Effects on Rapid Bimolecular Chemical Reactions. Chem. Rev..

[47] Collins F.C., Kimball G.E. (1949). Diffusion-controlled reaction rates. J. Colloid Sci..

[48] Das R., Periasamy N. (1989). Diffusion-Controlled Reactions: Analysis of Quenched Fluorescence Decay Data. Chem. Phys. Lett..

[49] Scully A.D., Hirayama S. (1995). Direct determination of kinetic parameters for diffusion-influenced reactions in solution by analysis of fluorescence decay curves. J. Fluoresc..

[50] Swallen S.F., Weidemaier K., Fayer M.D. (1995). Excluded Volume Effects in Photoinduced Electron Transfer and Geminate Recombination: Analytical Theory and Simulations. J. Phys. Chem..

[51] Matsuda N., Kakitani T., Denda T., Mataga N. (1995). Examination of the Viability of the Collins-Kimball Model and Numerical Calculation of the Time-Dependent Energy Gap Law of Photoinduced Charge Separation in Polar Solution. Chem. Phys..

[52] Murata S., Matsuzaki S.Y., Tachiya M. (1995). Transient Effect in Fluorescence Quenching by Electron Transfer. 2: Determination of the Rate Parameters Involved in the Marcus Equation. J. Phys. Chem..

[53] Shannon C.F., Eads D.D. (1995). Diffusion-controlled electron transfer reactions: subpicosecond fluorescence measurements of coumarin 1 quenched by aniline and *N*,*N*-dimethylaniline. J. Chem. Phys..

[54] Gladkikh V., Burshtein A., Tavernier H., Fayer M. (2002). Influence of Diffusion on the Kinetics of Donor-Acceptor Electron Transfer Monitored by the Quenching of Donor Fluorescence. J. Phys. Chem. A.

[55] Weller A. (1982). Photoinduced Electron Transfer in Solutions: Exciplex and Radical Ion Pair Formation Free Enthalpies and their Solvent Dependence. Z. Phys. Chem..

[56] Rozhkov I.N., Igumnov S.M., Bekker G.Y., Pletnev S.I., Rempel G.D., Deev L.E. (1989). Oxidative properties of perfluoroalkyl halides. Russ. Chem. Bull..

[57] Fukuzumi S., Okamoto T., Ohkubo K. (2003). Diels-Alder Reactions of Anthracenes with Dienophiles via Photoinduced Electron Transfer. J. Phys. Chem. A.

[58] Marcus R.A., Sutin N. (1985). Electron Transfer in Chemistry and Biology. Biochim. Biophys. Acta.

[59] Vauthey E. (2001). Direct measurements of the charge recombination dynamics of geminate ion pairs formed upon electron transfer quenching at high donor concentration. J. Phys. Chem. A.

[60] Rosspeintner A., Angulo G., Vauthey E. (2014). Bimolecular Photoinduced Electron Transfer Beyond the Diffusion Limit: The Rehm–Weller Experiment Revisited with Femtosecond Time Resolution. J. Am. Chem. Soc..

[61] Castner E.W., Kennedy D., Cave R.J. (2000). Solvent as Electron Donor: Donor/Acceptor Electronic Coupling is a Dynamical Variable. J. Phys. Chem. A.

[62] Angulo G., Cuetos A., Rosspeintner A., Vauthey E. (2013). Experimental Evidence of the Relevance of Orientational Correlations in Photoinduced Bimolecular Reactions in Solution. J. Phys. Chem. A.

[63] Rumble C.A., Vauthey E. (2019). Structural dynamics of an excited donor–acceptor complex from ultrafast polarized infrared spectroscopy, molecular dynamics simulations, and quantum chemical calculations. Phys. Chem. Chem. Phys..

[64] Burshtein A.I., Yakobson B.I. (1978). In-cage reactions controlled by molecular rotation. Chem. Phys..

[65] Gladkikh V.S., Burshtein A.I. (2007). Photoionization affected by chemical anisotropy. J. Chem. Phys..

[66] Cohen B., Huppert D., Agmon N. (2000). Non-Exponential Smoluchowski Dynamics in Fast Acid-Base Reaction. J. Am. Chem. Soc..

[67] Cohen B., Huppert D., Agmon N. (2001). Diffusion-Limited Acid-Base Nonexponential Dynamics. J. Phys. Chem. A.

[68] Rini M., Pines D., Magnes B.-Z., Pines E., Nibbering E.T. (2004). Bimodal proton transfer in acid-base reactions in water. J. Chem. Phys..

[69] Janiga A., Glodkowska-Mrowka E., Stoklosa T., Gryko D.T. (2013). Synthesis and Optical Properties of Tetraaryl-1,4-dihydropyrrolo[3,2-b]pyrroles. Asian J. Org. Chem..

[70] Gardecki J.A., Maroncelli M. (1998). Set of Secondary Emission Standard for Calibration of the Spectral Responsivity in Emission Spectroscopy. Appl. Spectrosc..

[71] Angulo G., Brucka M., Gerecke M., Grampp G., Jeannerat D., Milkiewicz J., Mitrev Y., Radzewicz C., Rosspeintner A., Vauthey E. (2016). Characterization of dimethylsulfoxide/glycerol mixtures: A binary solvent system for the study of “friction-dependent” chemical reactivity. Phys. Chem. Chem. Phys..

[72] Muller P.-A., Högemann C., Allonas X., Jacques P., Vauthey E. (2000). Deuterium Isotope Effect on the Charge Recombination Dynamics of Contact Ion Pairs Formed by Electron Transfer Quenching in Acetonitrile. Chem. Phys. Lett..

