# Supplementary material for: Halogen-Bond Assisted Photoinduced Electron Transfer
Source: Molecules. 2019 Nov 29;24(23):4361. doi: 10.3390/molecules24234361 (PMC6930453; doi:10.3390/molecules24234361)
Supplement: Supplementary file 1 [file molecules-24-04361-s001.pdf]

Supplementary Materials: Halogen-Bond Assisted Photoinduced Electron Transfer

Bogdan Dereka [†] 0000-0003-2895-7915, Ina Fureraj 0000-0001-8875-6344, Arnulf Rosspeintner 0000-0002-1828-5206 and Eric Vauthey* 0000-0002-9580-9683

Contents

1 Additional TRIR data 2

2 Additional time-resolved fluorescence data 3

3 Electrochemistry 6

4 Diffusion coefficients 6

1. Additional TRIR data

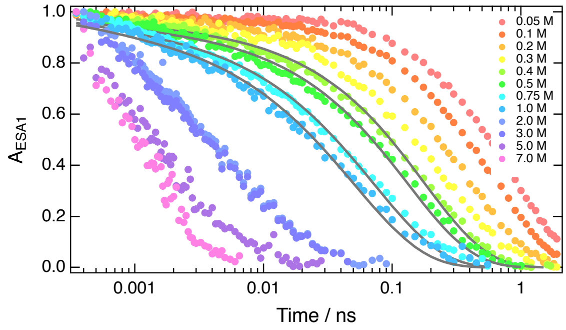

Figure S1. Excited-state decay of **ADA** measured by TRIR with different concentrations of HFIP in $CHCl_3$. The solid lines are the best fits of the SCK expression (Eq.2).

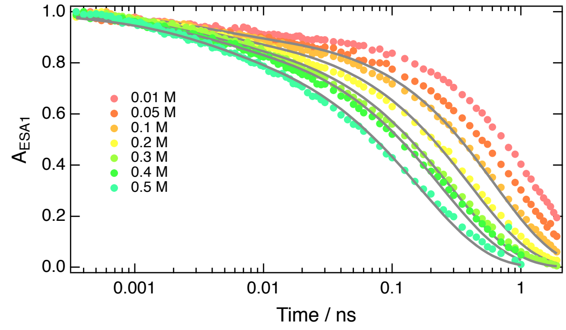

Figure S2. Excited-state decay of **ADA** measured by TRIR with different concentrations of HFIP in BCN. The solid lines are the best fits of the SCK expression (Eq.2).

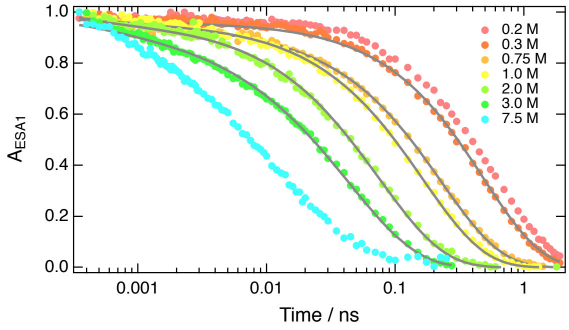

Figure S3. Excited-state decay of **ADA** measured by TRIR with different concentrations of IFB in $CHCl_3$. The solid lines are the best fits of the SCK expression (Eq.2).

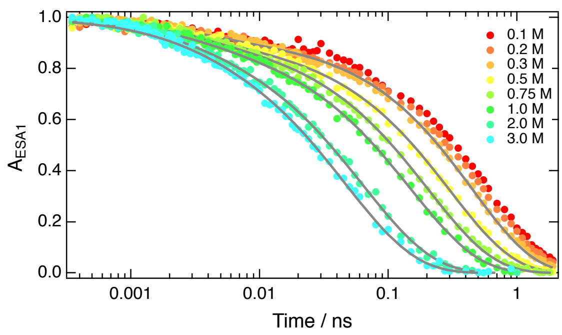

Figure S4. Excited-state decay of **ADA** measured by TRIR with different concentrations of IFB in BCN. The solid lines are the best fits of the SCK expression (Eq.2).

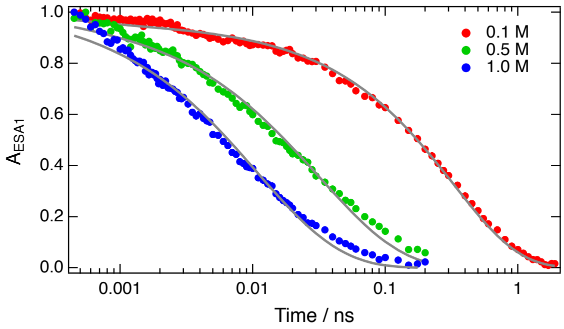

Figure S5. Excited-state decay of **ADA** measured by TRIR with different concentrations of FN in $CHCl_3$. The solid lines are the best fits of the SCK expression (Eq.2).

2. Additional time-resolved fluorescence data

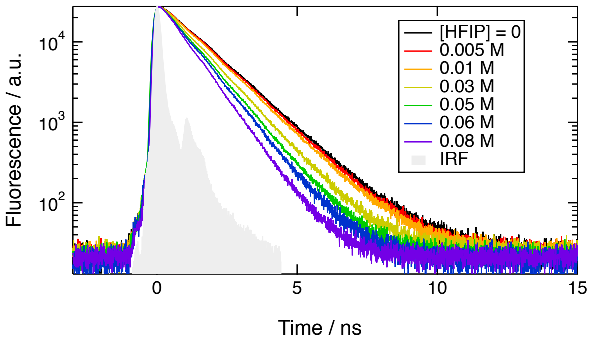

Figure S6. Fluorescence time profiles measured with **ADA** in $CHCl_3$ with different concentrations of HFIP.

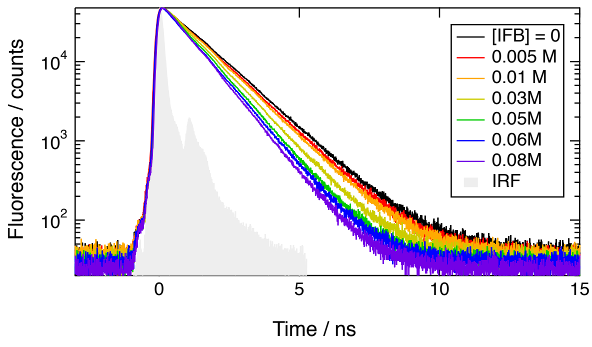

Figure S7. Fluorescence time profiles measured with **ADA** in $CHCl_3$ with different concentrations of IFB.

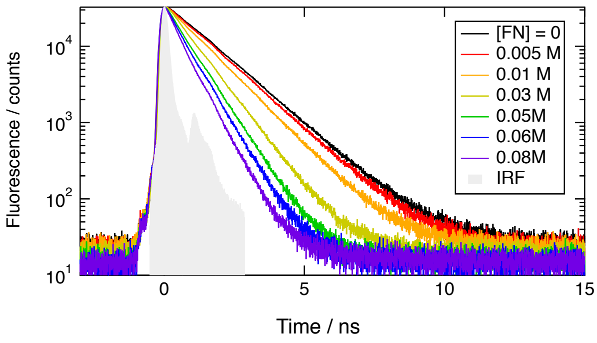

Figure S8. Fluorescence time profiles measured with **ADA** in $CHCl_3$ with different concentrations of FN.

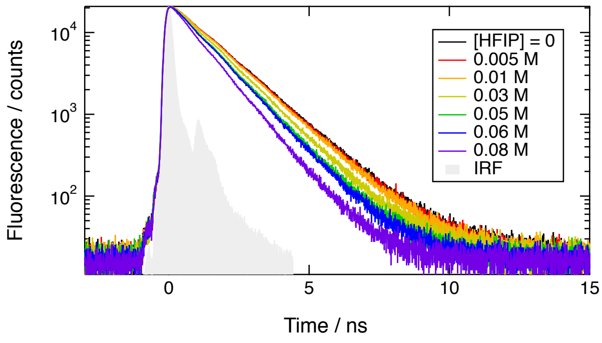

Figure S9. Fluorescence time profiles measured with **ADA** in BCN with different concentrations of HFIP.

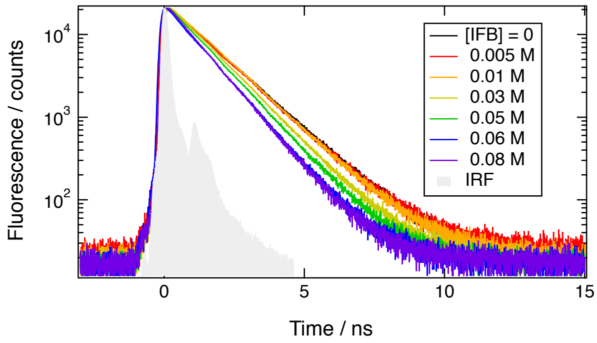

Figure S10. Fluorescence time profiles measured with **ADA** in BCN with different concentrations of IFB.

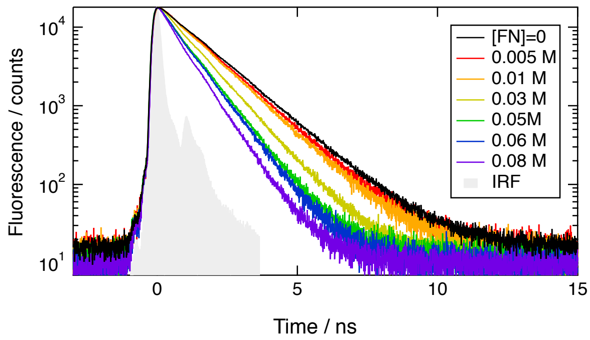

Figure S11. Fluorescence time profiles measured with **ADA** in BCN with different concentrations of FN.

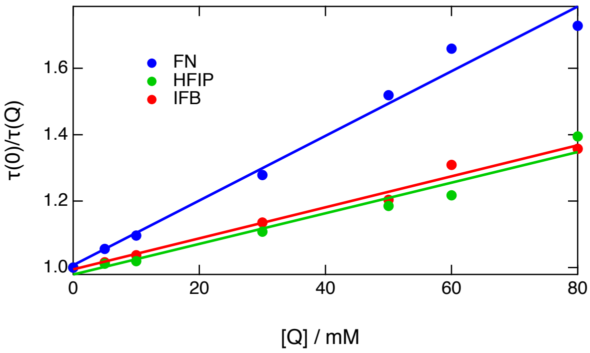

Figure S12. Stern-Volmer plots of the fluorescence quenching of **ADA** by HFIP, IFB and FN in BCN.

3. Electrochemistry

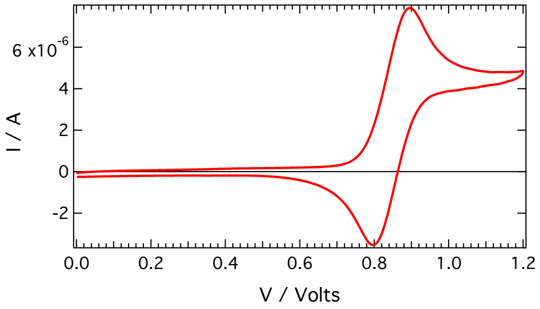

Figure S13. Cyclic voltammogram recorded with **ADA** in dichloromethane with tetrabutylammonium hexafluorophosphate as electrolyte using ferrocene as reference.

4. Diffusion coefficients

^{19}F NMR DOSY experiments were conducted to determine the diffusion coefficients of HFIP and IFB and ^{1}H NMR DOSY measurements for **ADA**. Two solvents were used: $CHCl_3$ and benzonitrile (BCN). Chloroform-d was used for the NMR to provide a deuterium lock, whereas non-deuterated benzonitrile-h5 was used with ^{19}F of added XB donor as a lock. NMR experiments for each chemical system were carried out with 4-5 solutions that were prepared as a function of molar fraction of the corresponding XB donor (x(HFIP) or x(IFB)). Those concentrations were (0), 25, 50, 75 and 100 molar % of the XB donor in a respective solvent. Ultrafast IR experiments were carried out on many solutions prepared with different molarities of donors. The conversion between molarity and molar fraction requires knowing the density of the solutions. The density was measured for solution with the same concentrations as used for the NMR. Additionally, density was computed based on the assumption of the ideal solution behaviour. No significant departure from ideal behaviour was noticed. The diffusion coefficients of **ADA** and XB donors were found to depend linearly on the concentration of XB donor in $CHCl_3$ and therefore measuring 4-5 solutions with DOSY NMR was sufficient to fit the observed dependence to a linear functional form which was used to assess the diffusion coefficients in each solution used for ultrafast experiments.
Due to the limited solubility of **ADA**, the D_{ADA} values could not be measured in BCN and were estimated by multiplying the D_{ADA} values measured in $CDCl_3$ by the $CDCl_3$/BCN viscosity ratio. The diffusion coefficient of FN was also measured up to 1M in $CDCl_3$. However, the D_{ADA} values at different FN concentrations were not determined, but were estimated by multiplying the D_{ADA} value in pure $CDCl_3$ by the $D_{FN}([Q])/D_{FN}(0)$ ratio, where $D_{FN}([Q])$ is the diffusion coefficient of FN at [Q], and $D_{FN}(0)$ is the value of D_{FN} extrapolated to [FN]=0. The resulting mutual diffusion coefficients are shown in Figures S14-S17 and Table S1.

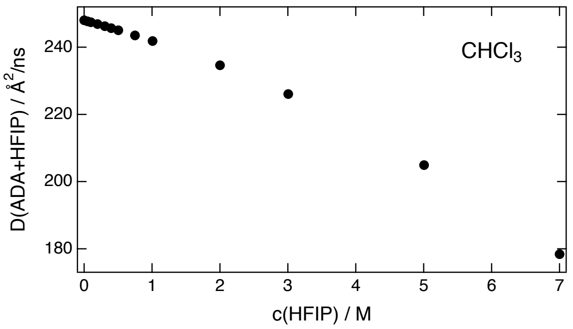

Figure S14. Mutual diffusion coefficients of **ADA** and HFIP in $CHCl_3$ at different HFIP concentrations.

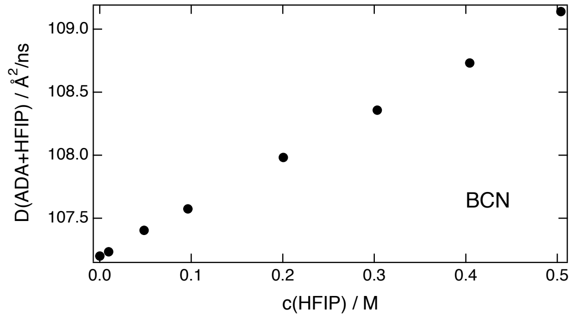

Figure S15. Mutual diffusion coefficients of **ADA** and HFIP in BCN at different HFIP concentrations.

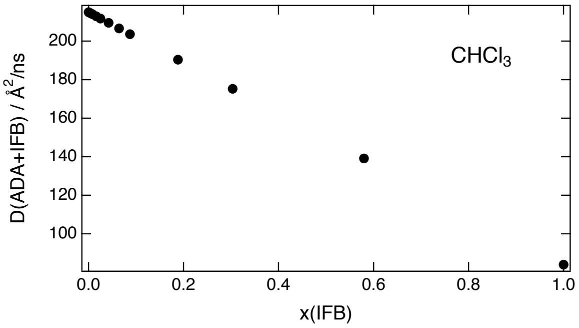

Figure S16. Mutual diffusion coefficients of **ADA** and IFB in $CHCl_3$ at different molar fraction of IFB in $CHCl_3$.

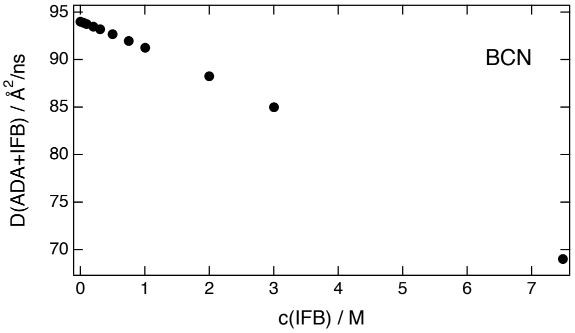

Figure S17. Mutual diffusion coefficients of **ADA** and IFB in BCN at different IFB concentrations.

Table S1. Mutual diffusion coefficient of **ADA** and FN in $CHCl_3$ at different FN concentrations.

**[FN]** / M	**D** / $M^{-1}ns^{-1}$
0.1	261
0.5	257
1	252
